# Supplementary material for: High Spatial but Low Temporal Variability in Ectomycorrhizal Community Composition in Abies alba Forest Stands
Source: Microorganisms. 2025 Jan 30;13(2):308. doi: 10.3390/microorganisms13020308 (PMC11858566; doi:10.3390/microorganisms13020308)
Supplement: Supplementary file 1 [file microorganisms-13-00308-s001.zip › microorganisms-3416270-supplementary.pdf]

Table S1: Diversity indices (richness, evenness, and dominance) through the season for sampling year 2016 and 2017 per individual sampling plot.

Location	Sampling year	Sampling month	Richness	Evennes	Dominance
Jelovški boršt		March	5 + 1.67	3.52 + 1.09	2.51 + 0.71
Jelovški boršt		April	5.83 + 1.33	3.34 + 0.63	2.33 + 0.63
Jelovški boršt		may	6.00 + 1.89	3.44 + 1.52	2.29 + 0.76
Jelovški boršt	2016	june	6.00 + 2.28	3.32 + 1.33	2.31 + 0.96
Jelovški boršt		july	3.83 + 2.13	2.90 + 1.43	2.40 + 1.12
Jelovški boršt		august	5.50 + 2.17	2.96 + 1.26	2.08 + 0.81
Jelovški boršt		september	4.67 + 1.21	2.82 + 0.53	2.06 + 0.44
Jelovški boršt		october	3.67 + 2.06	2.98 + 1.66	2.38 + 1.19
Jelovški boršt		november	3.67 + 2.16	2.25 + 1.03	1.79 + 0.60
Lehen na Pohorju		march	3.00 + 1.41	1.93 + 0.93	1.53 + 0.58
Lehen na Pohorju		april	5.67 + 1.97	3.58 + 1.05	2.64 + 0.76
Lehen na Pohorju		may	3.17 + 1.17	2.17 + 0.47	1.72 + 0.31
Lehen na Pohorju		june	3.50 + 1.22	2.18 + 0.85	1.71 + 0.61
Lehen na Pohorju		july	3.83 + 1.47	2.64 + 1.07	1.95 + 0.53
Lehen na Pohorju	2016	august	3.17 + 0.98	1.84 + 0.86	1.52 + 0.66
Lehen na Pohorju		september	2.83 + 0.98	1.94 + 0.65	1.53 + 0.36
Lehen na Pohorju		october	3.33 + 1.03	2.27 + 0.63	1.74 + 0.47
Ljubelj		march	4.00 + 2.37	2.38 + 0.98	1.80 + 0.56
Ljubelj		april	4.00 + 1.26	2.63 + 0.69	2.02 + 0.45
Ljubelj		may	3.00 + 0.89	1.96 + 0.68	1.58 + 0.49
Ljubelj		june	3.50 + 1.05	1.90 + 0.88	1.56 + 0.67
Ljubelj		july	3.00 + 1.55	2.20 + 0.81	1.78 + 0.56
Ljubelj		august	2.00 + 0.63	1.42 + 0.35	1.22 + 0.17
Ljubelj		september	2.67 + 0.82	2.01 + 0.52	1.75 + 0.41
Ljubelj		october	2.17 + 1.17	1.33 + 0.42	1.18 + 0.24
Jelovški boršt	2017	march	4.67 + 1.37	2.78 + 0.88	2.05 + 0.67
Jelovški boršt		april	3.17 + 1.94	2.11 + 0.89	1.73 + 0.61
Jelovški boršt		may	4.17 + 1.72	3.22 + 1.46	2.35 + 0.83
Jelovški boršt		june	4.00 + 1.67	2.58 + 1.12	1.96 + 0.70
Jelovški boršt		july	3.60 + 1.82	2.47 + 1.47	1.91 + 0.85
Jelovški boršt		august	2.50 + 1.52	1.57 + 0.50	1.38 + 0.39
Jelovški boršt		september	3.83 + 1.83	2.18 + 0.82	1.64 + 0.46
Jelovški boršt		october	3.67 + 1.96	2.02 + 1.13	1.54 + 0.64
Lehen na Pohorju	2017	march	2.50 + 1.22	1.62 + 0.49	1.38 + 0.32

Lehen na Pohorju		april	2.17 + 1.17	1.81 + 0.79	1.67 + 0.72
Lehen na Pohorju		may	2.17 + 1.33	1.56 + 0.66	1.29 + 0.33
Lehen na Pohorju		june	2.33 + 0.52	1.61 + 0.36	1.41 + 0.33
Lehen na Pohorju		july	2.50 + 1.22	1.89 + 1.00	1.63 + 0.83
Lehen na Pohorju		august	2.00 + 0.63	1.47 + 0.56	1.28 + 0.36
Lehen na Pohorju		september	3.50 + 1.73	2.64 + 1.13	2.04 + 0.75
Lehen na Pohorju		october	3.00 + 1.67	1.74 + 0.59	1.45 + 0.42
Ljubelj	2017	march	4.00 + 2.28	2.86 + 1,25	2.23 + 0.84
Ljubelj		april	3.00 + 1.67	1.94 + 0.59	1.60 + 0.59
Ljubelj		may	4.00 + 1.79	2.54 + 1.25	1.91 + 0.78
Ljubelj		june	2.83 + 0.98	1.59 + 0.49	1.33 + 0.31
Ljubelj		july	3.00 + 0.70	2.17 + 0.62	1.81 + 0.59
Ljubelj		august	2.83 + 1.33	2.18 + 1.18	1.72 + 0.73
Ljubelj		september	3.17 + 1.47	2.11 + 1.04	1.69 + 0.80
Ljubelj		october	3.50 + 1.38	2.17 + 1.08	1.63 + 0.58

Table S2: Generalized linear models results (GLMs) for ECM fungal taxon, that occur rarely at individual study site and for which statistically significant different abundance was confirmed along to sampling month. Different marks (*) shows significantly different results (GLM, p < 0,05 *; p < 0,01 **; p < 0,001 ***).

ECM taxa at site Jelovški boršt	P-value	ECM taxa at site Ljubelj	P-value	ECM taxa at site Lehen na Pohorju	P-value
Amanita rubescens	0.0068**	*Cantharellales* sp.	6.492e-05***	*Cortinarius decipiens*	0.0132*
Sutorius luridiformis	0.0470*	*Cortinarius decipiens*	0.0119*	*Elaphomyces asperulus*	0.0133*
Hebeloma theobrominum	0.00414**	*Craterellus tubaeformis*	0.0119*	*Lactifluus lignyotus*	0.0129*
Inocybe assimilata	0.0075**	*Russula amtehystina*	0.0119*	*Russula illota*	0.0135*
Inocybe fuscidula	0.0123*	*Russula delica*	0.0016**	*Russula integra*	0.0126*
Laccaria amethystina	0.0006***	*Russula fellea*	0.0355*	*Imleria badia*	0.0164*
Lactifluus volemus	4.026e-05***	*Russula illota*	0.0119*	*Xerocomellus chrysenteron*	0.0009***

Russula delica	0.0100**	*Russula* sp.	0.0119*		
Russula queletii	0.0101*	*Tylospora fibrillosa*	0.0123*		
Terfezia sp	0.0037**				
Tomentella badia	0.0101*				
Tricholoma virgatum	0.0007***				

Table S3: Analysis of sampling month influence on most abundant ECM taxon, identified at specific study site. ECM taxon, that appeared at specific study site for at least 10x through two years of sampling in at least 5% of all soil samples were included in the analyses (GLM with ANOVA test, p < 0,05 *; p < 0,01 **, p < 0,001 ***).

ECM taxa | Study site	Jelovški boršt	Ljubelj	Lehen na Pohorju
Amanita rubescens	/	0.0003***	0.0122*
Amphinema byssoides	0.257	0.6619	0.057
Neoboletus erythropus	/	0.0689	0.0034 **
Cenococcum geophilum	0.9342	0.3924	0.548
Clavulina corraloides	0.4869	0.00316**	0.4757
Elaphomyces granulatus	/	/	6.553e-06***
Lactarius salmonicolor	0.148	0.1577	0.0078**
Lactarius subdulcis	0.4647	/	5.197e-05***
Russula cyanoxantha	0.0001***	0.0630	0.4757
Russula chloroides	0.4647	0.0112*	/
Russula badia	/	/	0.223
Russula illota	0.0282*	0.0119*	/
Russula nigricans	0.0188*	0.0069**	0.4757
Russula ochroleuca	0.5900	0.0279*	0.0385*
Russula turci	0.1831	0.0103*	/
Sebacina epigeae	2.482e-05***	0.0359*	1.976e-10***
Sebacina incrustans	0.14	0.2743	/
Tomentella stuposa	0.189	0.0818	0.0001***
Thelephora wakefieldiae	0.0463*	0.5936	0.0226*
Tricholoma virgatum	0.0007***	0.0102*	0.0224*

Tylospora fibrillosa	0.1466	0.0123*	0.6839
Xerocomellus pruinatus	/	0.4635	1.776e-05***
